# Nosocomial Outbreak of Extensively Drug-Resistant (Polymyxin B and Carbapenem) *Klebsiella pneumoniae* in a Collapsed University Hospital Due to COVID-19 Pandemic

**DOI:** 10.3390/antibiotics11060814

**Published:** 2022-06-17

**Authors:** Gilberto G. Gaspar, Gustavo Tamasco, Nathália Abichabki, Ana Flavia T. Scaranello, Maria Auxiliadora-Martins, Renata Pocente, Leonardo N. Andrade, María-Eugenia Guazzaroni, Rafael Silva-Rocha, Valdes R. Bollela

**Affiliations:** 1 Ribeirão Preto School of Medicine (FMRP), University of São Paulo (USP), Av. Bandeirantes 3900, Ribeirão Preto 14049-900, SP, Brazil; gustavo.tamasco@usp.br (G.T.); silvarochar@usp.br (R.S.-R.); vbollela@fmrp.usp.br (V.R.B.); 2 School of Pharmaceutical Sciences of Ribeirão Preto (FCFRP), University of São Paulo (USP), Av. Bandeirantes 3900, Ribeirão Preto 14049-900, SP, Brazil; nathalia.abichabki@alumni.usp.br (N.A.); leonardo@fcfrp.usp.br (L.N.A.); 3 Faculty of Philosophy, Sciences and Letters of Ribeirão Preto (FFCLRP), University of São Paulo (USP), Av. Bandeirantes 3900, Ribeirão Preto 14049-900, SP, Brazil; anatonelli@usp.br (A.F.T.S.); meguazzaroni@ffclrp.usp.br (M.-E.G.); 4 Clinics and University Hospital from Ribeirão Preto School of Medicine (FMRP), University of São Paulo (USP), Av. Bandeirantes 3900, Ribeirão Preto 14049-900, SP, Brazil; mamartins@hcrp.usp.br (M.A.-M.); rhcpocente@hcrp.usp.br (R.P.)

**Keywords:** clinical evaluation, polymyxin resistance, extensively drug-resistance, multidrug-resistance, molecular epidemiology, KPC

## Abstract

We correlated clinical, epidemiological, microbiological, and genomic data of an outbreak with polymyxin B (PB)- and carbapenem-resistant *Klebsiella pneumoniae* during the COVID-19 pandemic. Twenty-six PB- and carbapenem-resistant *K. pneumoniae* were isolated from patients in the COVID-19 ICU (Intensive Care Unit), non-COVID-19 ICU (Intensive Care Unit), clinical, or surgical ward. Bacterial identification, drug susceptibility tests, and DNA sequencing were performed, followed by in silico resistance genes identification. All isolates showed extensively drug-resistant (XDR) phenotypes. Four different sequence types (ST) were detected: ST16, ST11, ST258, and ST437. Nineteen isolates were responsible for an outbreak in the ICU in September 2020. They belong to ST258 and harbored the 42Kb IncX3plasmid (pKP98M3N42) with the same genomic pattern of two *K. pneumoniae* identified in 2018. Twenty-four isolates carried *bla*_-KPC-2_ gene. No plasmid-mediated colistin (*mcr*) resistance genes were found. Eight isolates presented *mgrB* gene mutation. The clonal isolates responsible for the outbreak came from patients submitted to pronation, with high mortality rates in one month. XDR-*K. pneumoniae* detected during the outbreak presented chromosomal resistance to PB and plasmid-acquired carbapenem resistance due to KPC production in most isolates and 42Kb IncX3(pKP98M3N42) plasmid carrying *bla*_KPC-2_ was associated with ST258 isolates. The outbreak followed the collapse of the local healthcare system with high mortality rates.

## 1. Introduction

The first cases of COVID-19 in Brazil were reported on 25 February 2020. One year later, more than half a million deaths were reported due to COVID-19, most of them after a long stay in intensive care units (ICU). Severe cases were described to be frequently associated with comorbidities, age above 50, exacerbated inflammatory response, and invasive procedures (mechanical ventilation, central intravenous access), which facilitated healthcare-associated infections (HAI) [1]. Hospitalized patients with COVID-19 and bacterial secondary infections have commonly been detected, resulting in high rates of antibiotic use (up to 94 to 100%) among patients with confirmed cases of COVID-19 [2]. Several studies showed an increase in antimicrobial resistance during the COVID-19 pandemic, mainly related to Gram-negative bacilli, driven by abusive antimicrobial use, overload of health professionals, and prolonged time with invasive therapy.

Antimicrobial resistance during the COVID-19 pandemic is highly associated with the presence of *Acinetobacter baumannii* and *Klebsiella pneumoniae*, mainly carbapenemase-producing (e.g., KPC) isolates [3,4]. A review evaluating the impact of the ongoing pandemic on antimicrobial resistance showed that the vast majority of the reports came from ICU. Reports of clusters of infections due to the same resistant pathogen raised the concern of a decline in adherence to infection control and prevention measures. Another aspect is the importance of environmental contamination [4].

A previous study in our hospital, from 2018 to 2020, showed an increase in the rate of *A. baumannii* and *K. pneumoniae* resistant to carbapenems per 1000 patients/day after the beginning of the COVID-19 pandemic. There was a 10-fold increase (from 5% to 50%) in the number of polymyxin B (PB)-resistant *K. pneumoniae* isolation comparing 2018–2019 (before COVID-19) to the same period after the pandemic onset [5].

This study aimed to characterize and correlate clinical, epidemiological, microbiological, and genomic data from an outbreak of PB- and carbapenem-resistant *K. pneumoniae* isolated from patients in a tertiary hospital during the collapse of the healthcare system in 2020 due to the COVID-19 pandemic.

## 2. Materials and Methods

### 2.1. Study Design

We included 26 PB- and carbapenem-resistant *K. pneumoniae* isolated from patients in a hospital with 551 beds just after healthcare collapse due to COVID-19. Patients were admitted into one of the four medical units: COVID-19 ICU, non-COVID-19 ICU, clinical, or surgical ward. The genomic profile of 2020 *K. pneumoniae* detected during COVID-19 pandemic were compared with isolates sequenced in 2018 in the same hospital, which is the reference for 3.5 million people in Southern Brazil (Ribeirão Preto region of São Paulo State).

This study was approved by the Ethics Committee of the Hospital das Clínicas da Faculdade de Medicina de Ribeirão Preto-Universidade de São Paulo (CAAE: 51569821.9.0000.5440).

### 2.2. Clinical Data and Context

Clinical data were collected from 1 September to 30 November 2020. COVID-19′s first peak occurred from June to August 2020 in Brazil. The following information was collected: gender, age, underlying disease, SARS-CoV-2 infection, hospitalization time, HAI, hospital unit where *K. pneumoniae* were isolated, previous use of polymyxin B (PB), and other antibiotics. The clinical samples were diagnostic of hospital infection, and the samples included in the study were: blood, tracheal secretion, urine, peritoneal fluid, and surgical wound.

### 2.3. Identification and Antimicrobial Susceptibility Testing of Isolates

The identification of bacterial isolates and the antimicrobial susceptibility testing were performed by Vitek^®^2 (bioMérieux, Marcy l’Etoile, France). Ceftazidime-avibactam (CZA) susceptibility using ETEST^®^ (bioMérieux, Marcy l’Etoile, France) or by standardized disk diffusion method. PB minimum inhibitory concentration (MIC) was first determined using the commercial test based on a panel with lyophilized PB, Policimbac^®^ (Probac, São Paulo, Brazil) [6].

PB MIC was confirmed using the reference method of broth microdilution assay [7]. Water solution of PB (United States Pharmacopeia, Rockville, United States of America), Cation-Adjusted Mueller Hinton II Broth (CAMHB) (BBL™, Becton Dickinson, New Jersey, United States of America), and 96-well microplates polystyrene, round bottom, non-treated, were used for assays. Two-fold serial dilution (256–0.5 µg/mL) of PB was evaluated and MIC values were determined as the lowest concentrations of PB that inhibit visible bacterial growth in CAMHB. *Escherichia coli* ATCC 25,922 and *E. coli* NCTC 13,846 (*mcr-1* positive) were used as quality control. The strains were considered PB-resistant when MIC was higher than 2 mg/L, according to EUCAST [7].

### 2.4. DNA Extraction and Genome Sequencing

Total genomic DNA was extracted using the Wizard Genomic DNA Purification Kit (Promega, Madison, WI, USA) following the manufacturer’s instructions. The DNA concentrations were measured fluorometrically (Qubit^®^ 3.0, kit Qubit^®^ dsDNA Broad Range Assay kit, Life Technologies, Carlsbad, CA, USA). Purified DNA was prepared for sequencing using the Nextera XT DNA Library Prep Kit (Illumina, San Diego, CA, USA). Libraries were assessed for quality using the 2100 Bioanalyzer (Agilent Genomics, Santa Clara, CA, USA). DNA sequencing was performed at Novogene (Sacramento, CA, USA) on a Novaseq6000 platform. Adapters were trimmed using Trimmomatic v0.36. Samples were evaluated for possible contamination using Bowtie2 v2-2.2.3. Overlapped reads were merged using Flash version 1.2.11. Merged and unmerged reads were assembled using Spades v3.12.0. Genome quality (completeness and contamination) was evaluated using CheckM v1.0.7 and QUAST. Genome annotations were performed using Prokka v1.11 with default parameters. The amino acid sequences of all genes identified, using Prokka, were aligned with the National Database of Antibiotic Resistant Organisms (NDARO) database obtained from NCBI (March 2021).

The alignment was performed using Diamond v0.8.24. Alignments with ≥60 similarity score were selected for further analysis. Final quality of genome assembly was determined using MIGA web service. Only samples with High Quality (>80%), High Level of Completeness (>95%), and Low Contamination (<3%) were selected for further analysis. All genomes are available at the NCBI under the BioProject number PRJNA755436.

### 2.5. In Silico Identification of Carbapenem and Polymyxin B Resistance Genes and Characterization of Bacterial Genomic Relationship

The identification of acquired carbapenemase resistance genes was performed using the ABRICATE pipeline by searching annotated genes using reference databases (ARG-ANNOT, NCBI AMRFinderPlus, CARD, and ResFinder). Chromosomal and plasmid localization of resistance genes was evaluated using ARG information from ARG-ANNOT [8]. Identification of plasmid-carrying carbapenemase genes was performed using PlasmidFinder (https://cge.cbs.dtu.dk/services/PlasmidFinder/ (accessed on 19 March 2021).

Chromosomal genes related to PB resistance were also investigated [3,9,10,11]. Genomes of PB-resistant *K. pneumoniae* clinical isolates studied were compared to the genome of PB-susceptible and KPC-producing *K. pneumoniae* ATCC BAA-1705 standard strain (https://www.patricbrc.org/view/Genome/1276652.3 (accessed on 30 April 2021). Mutations were manually evaluated and annotated for each clinical isolate studied.

Phylogenomic analyses were performed using Parsnp and Gingr. Phylogenomic trees were produced by iTOL. Data manipulation was completely performed in Python (3.7.4), all scripts can be found in the git repository (https://github.com/tamascogustavo/project_MDR_KRP (accessed on 31 August 2021). Sequence type (ST) determination was carried out in the *Klebsiella* Multi-Locus Sequence Typing (MLST) database, from Institute Pasteur, based on whole genome sequence [12].

## 3. Results

### 3.1. K. pneumoniae Clinical Isolates Features

**Figure 1:**
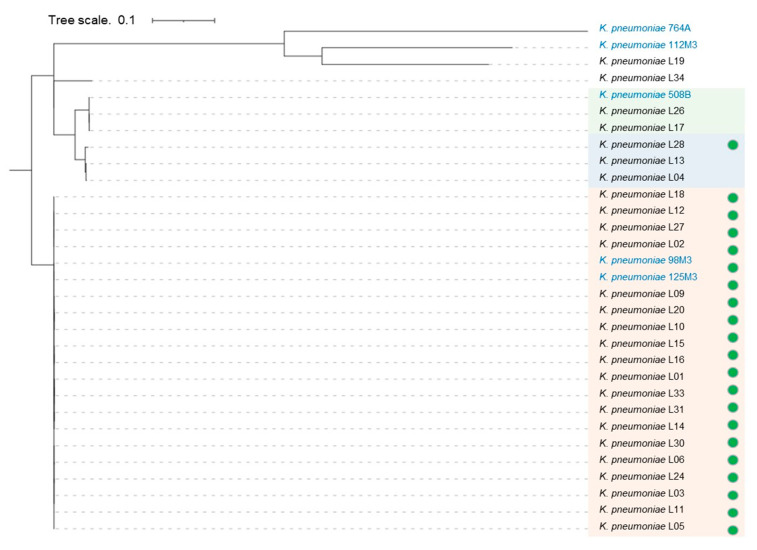
Phylogenomic tree of 26 *K. pneumoniae* isolated in 2020 (black) during the COVID-19 pandemic, with five isolates sequenced in 2018 in the same hospital (light blue), based on SNP detection. Isolates from the “orange group” belong to ST258 and isolates from the “green and blue groups” belong to the ST11. Bacteria holding plasmid pkP98M3N42 with coverage scores over 90% and similarity scores over 99% with the reference [15] are indicated with a green solid circle.

Based on the SNP’s analysis, four groups were detected, and they were related to the sequence types ST11, ST16, ST258, and ST437 with a predominance of the clonal group (CG) 258 (Figure 1). All the isolates were extensively drug-resistant (XDR) [13], as shown in Table S1.

Among the 26 *K. pneumoniae* initially detected as PB-resistant, 20 were confirmed by broth microdilution assay. No plasmid-mediated colistin (*mcr*) resistance genes were found. Eight isolates (30.8%) presented mutation in *mgrB* gene, *pmrA* gene mutation was found in most isolates (96.1%) except L14, and none presented mutations in *phoP, phoQ,* or *pagP* genes (Table 1). Mutations in those genes result in the addition of cationic groups in the lipopolysaccharide (LPS), leading to the repulsion of polymyxin molecule, which results in polymyxin resistance [14].

*K. pneumoniae* L13 and L04 isolates belong to the same ST11 clone (Table 1 and Figure 1). They presented resistance to ertapenem (ERT), intermediate resistance (susceptible with increased exposure) to meropenem (MER), and susceptibility to imipenem (IPM). Only these two isolates did not harbor the *bla*_KPC-2_ gene (or another carbapenemase gene). Additionally, L13 and L04 isolates showed different susceptibility to PB (respectively, MIC = 32 µg/mL, and MIC = ≤ 0.5 µg/mL) as well as different mutation in genes related to PB susceptibility (respectively, *mgrB*, *pmrA*, *pmrD*, and *pmrA*, *pmrD*, *ramA*).

The 42 Kb IncX3 plasmid (pKP98M3N42) was identified on 19/26 (73%) isolates from ST258 and in L28 isolate belonging to ST11, harboring *bla*_KPC-2_ resistance determinant (coding for *K. pneumoniae* carbapenemase [KPC], a beta-lactamase that may confer resistance to all beta-lactam antibiotics, highlighted carbapenems) (Figure 1). In addition, transposases, recombinases, and type IV secretion system genes were found in this plasmid [15].

Other different resistance determinants were found, including broad spectrum and extended spectrum beta-lactamase (ESBL) genes (*bla*_SHV_ and *bla*_CTX-M_), plasmid-mediated quinolone resistance gene (*qnr*), fosfomycin resistance gene (*fos*), aminoglycoside modifying enzymes (AMEs), and 16S rRNA methylases enzymes resistance genes (*rmt, aac, aad, ant*), and tetracyclines resistance gene (*tet*) (Table S2 and Figure S1).

### 3.2. Clinical Features and Patients’ Outcomes

**Figure 2:**
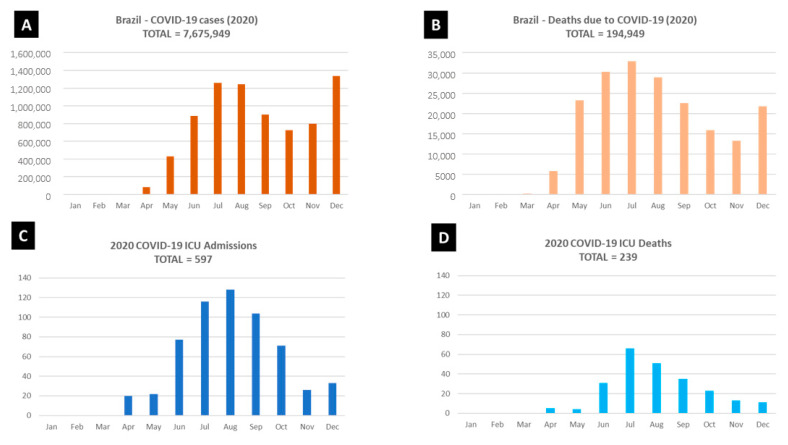
Number of COVID-19 cases (**A**) and deaths (**B**) in Brazil during 2020. Number of hospital intensive care unit (ICU) admissions (**C**) and deaths (**D**) at COVID-19 ICU in 2020. Source: Brazil: Coronavirus Pandemic Country Profile-Our World in Data-https://ourworldindata.org/coronavirus/country/brazil (accessed on 30 March 2022).

The number of COVID-19 patients and ICU beds in the hospital increased exponentially after March 2020. The number of patients in the COVID-19 ICU increased from zero (March) to 128 (August 2020) (Figure 2). Concurrently, the number of COVID-19 ICU beds was increased from the initial 21 to 89 in four ICUs in the same period. The number of *K. pneumoniae* resistant to PB isolated in the hospital raised from 100 in 2019, to 350 (2020) and 450 till August 2021 (Figure S2).

The incidence density for PB-resistant *K. pneumoniae* also demonstrates an increase in the pandemic period related to the pre-pandemic period (18.5 positive samples for PB-resistant *K. pneumoniae*/1000 patients/day versus 1.5 positive samples for PB-resistant *K. pneumoniae*/1000 patients/day). The sites of infection are described in Table 2. Eight (30.8%) *K. pneumoniae* in the study were considered colonization. In the other 18 patients (69.2%) *K. pneumoniae* was the cause of HAI.

Most (80%) patients who died had at least one comorbidity with cardiovascular diseases being predominant. Among 26 selected patients, eight (30.7%) died: five in September, two in October, and two in November. All the patients received antibiotics treatment that included one or more of the following: azithromycin (previous use of antimicrobial during hospitalization before isolation of the extensively drug-resistant *Klebsiella pneumoniae*.) ceftriaxone, cefepime, piperacillin-tazobactam, aminoglycosides, and carbapenem.

**Figure 3:**
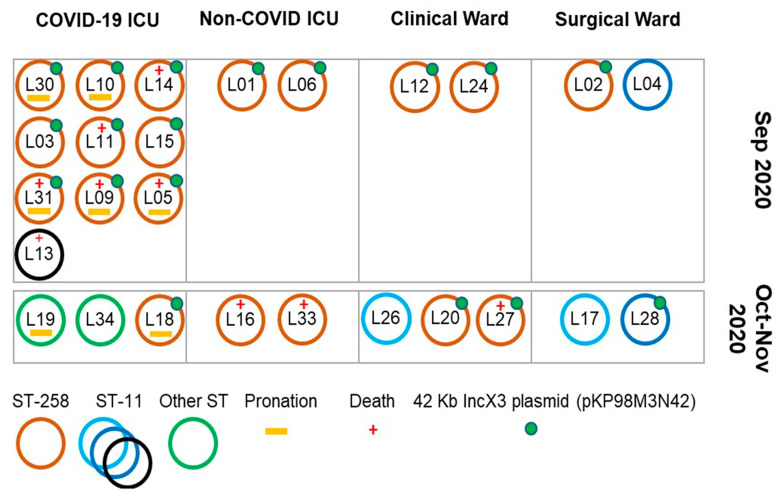
Schematic representation of the distribution of the 26 *K. pneumoniae* isolates recovered from hospitalized patients from September to November 2020 in the COVID-19 ICU, Non-COVID-19 ICU, Clinical Ward, and Surgical Ward. Legends represent the color code for the sequence type (ST), presence of 42 Kb IncX3 plasmid (pKP98M3N42) harboring *bla*_KPC-2_ (green solid circle), pronation practices in hospitalized patients (orange line), and death of the patient (red cross).

Two-thirds (16) of PB- and carbapenem-resistant *K. pneumoniae* isolates were detected in September 2020 and ten (38%) in October and November 2020 (Figure 3). The COVID-19 first wave peak in Southern Brazil occurred from June to August 2020, when the healthcare system collapsed for the first time (Figure 2).

In September 2020, 17 PB- and carbapenem-resistant *K. pneumoniae* were isolated in the hospital, and most of them, 11 (64.7%), were recovered from patients in the COVID-19 ICU. During this month, 15 out of 17 (88.2%) isolates were defined as ST258, with nine patients in the COVID-19 ICU, two in non-COVID ICU, two in the clinical, and two in the surgical ward. Six out of 11 (54.5%) COVID-19 patients in the ICU died, and five (80%) were from ST258. Fifty percent of the ST258 isolates were from patients who required pronation during COVID-19 management in the ICU. Six out of 10 (60%) patients with *K. pneumoniae* ST258 infections died in the COVID-19 ICU during September (Figure 3 and Table 2).

From October to November 2020, a homogeneous distribution of *K. pneumoniae* infection cases could be observed among clinical settings. ST258 was the predominant lineage with 50% of the cases (Table 1). Three deaths were observed during this period, two in the non-COVID ICU and one in the clinical ward (Figure 3).

In this period, there was an increase in healthcare-related infection rates, the main ones being ventilator-associated pneumonia and primary bloodstream infection (Table 2). The HAI was computed among patients with KPC infection. Figure 2B lists the COVID-19 deaths in the year 2020 in Brazil.

## 4. Discussion

Our investigation of the nosocomial outbreak of extensively drug-resistant *K. pneumoniae* during the COVID-19 pandemic revealed a concerning scenario. The rapid spread of SARS-CoV-2 and the increased number of severe cases resulted in the system’s collapse.

An overcrowded ICU, lack of specialized healthcare professionals, and other healthcare resources during the peak of COVID-19 aggravated the situation. Tiri et al. (2020) observed an increase in the number of Carbapenem-resistant *Enterobacteriaceae* (CRE) at the University Hospital of Terni (Italy). Analyzing patients colonized with CRE on a quarterly basis, the authors observed the incidence of CRE from 6.7% in 2019 to 50% in March/April 2020, at the peak of the COVID-19 pandemic. High workload of professionals, prone position, lack of professional experience in the ICU setting, and overuse of antimicrobials may have accounted for this increase during the pandemic [16]. The same was observed in our study in the COVID-19 ICUs during September 2020.

Adebsi et al. (2021) had already evidenced that secondary bacterial infections play crucial roles in mortality and morbidity associated with COVID-19 [17]. The challenge to differentiate bacterial- from COVID-19 pneumonia and the absence of antiviral therapy with confirmed efficacy and the urgent need to start some therapy increase the use of antibiotics as part of the strategy to manage COVID-19 critical patients [18]. A study in Peru reported that 68.9% of patients with COVID-19 received antibiotics (mainly azithromycin and ceftriaxone), and a self-medication rate of 33%, before hospital admission [19].

XDR clinical isolates, especially co-resistant to carbapenems, aminoglycosides, polymyxins (e.g., PB), and tigecycline (CAPT-resistant) have been increasingly reported worldwide and represent a real possibility of dissemination of pandrug-resistant Gram-negative bacilli. KPC-2-producing *K. pneumoniae* CG258 (ST258 and ST11 international high-risk clones, as well as ST437) have been circulating all over the world and in our hospital, at least, since 2007 initially represented by a clonal outbreak of ST258 [20,21,22].

PB-resistant and KPC-2-producing *K. pneumoniae* ST11 emerged and was firstly detected in 2012. These isolates were characterized by a clonal outbreak that also contained the same ST11 clone susceptible to PB, showing an initial diversification and selection of PB-resistant ST11 clones in our hospital.

The outbreak isolates were compared to previous *K. pneumoniae* identified in the same hospital two years prior and two (98M3 and 125M3) belong to ST258 which harbors the 42 Kb IncX3 plasmid (pKP98M3N42), with the exact pattern observed in 19 *K. pneumoniae* that was responsible for the outbreak in the COVID-19 ICU in September 2020 [10]. *K. pneumoniae* isolate L28 also harbors 42 Kb IncX3 plasmid but belongs to a different ST (ST11; blue group); however, it is part of the same clonal group (CG258) shared by the 19 *K. pneumoniae* isolates gathered in the orange group (Figure 1).

The CG258 clonal group includes ST258, ST11, ST340, ST437, and ST512 [23], and it is related to the acceleration of spread in nosocomial outbreaks when associated with the resistance gene, which is comprised in the pKP98M3N42 plasmid [24].

This epidemiological scenario is similar worldwide and the association of the gene *bla*_KPC-2_ and CG258 isolates have boosted the outbreaks as well as endemicity of these bacteria [25].

We observed five infections due to ST11 isolates resulting in three deaths. Jin et al. (2021) analyzed 11 isolates of carbapenem-resistant *K. pneumoniae* with a *bla*_KPC-2_ located in a same size plasmid. This is a potential threat to anti-infective treatment and is capable of rapid and diverse evolution of resistance during treatment with tigecycline and polymyxin [26]. Mutations associated with the *mgrB* gene, detected in eight isolates in this study (Table 1), are the most common cause of polymyxin resistance in *K. pneumoniae* [27,28].

Although mutations in the *pmrA* gene were detected in most of the 26 isolates, it was described that only a few mutations on this gene are responsible for PB resistance [28]. This might explain why some isolates with mutations in *pmrA* were susceptible to PB (Table 1).

The 42 Kb IncX3 plasmid (pKP98M3N42), harboring *bla*_KPC-2_, has also been reported in bacterial strains in Brazil since 2009 [15] and plays an important role in horizontal gene transfer conferring resistance to carbapenems [23] and polymyxins [10].

Interesting, *K. pneumoniae* L13 and L04 isolates belong to the same ST11 clone and showed different PB susceptibility, mutations in genes related to PB susceptibility, and different dates and places of isolation in the hospital. Thereby, these characteristics and scenarios suggest intra-clonal diversification and evolution of the L04 isolate (mutation in *mgrB* gene and PB MIC = 32 µg/mL) (Table 1).

From July to September 2020, COVID-19 ICU had 100% occupancy, despite the number of beds increasing more than four times. These new ICU places were created due to the high number of COVID-19 critical cases. In September 2020, a likely association can be observed between high mortality and infections due to PB- and carbapenem-resistant *K. pneumoniae* ST258, carrying 42Kb-IncX3-*bla*_KPC-2_ in the COVID ICU. In the following two months, the distribution of cases among clinical wards changed and the number of isolates decreased significantly; however, ST258 was still predominant.

As several isolates are identical by genomic sequencing, we can assume (hypothesis) that this isolate has increased in frequency in the ICU COVID because of the difficulties of treatment and control of the isolate. As there are patients who are discharged from the ICU to wards, this isolate may have been taken to these other places, or the other possibility is that due to the overload of work faced by the teams, many doctors and nurses were required to cover more than one unit in a rotation scheme, moving them between different units during the worst period of the pandemic (Figure 3).

This study has some limitations as we analyzed only positive clinical samples with *K. pneumoniae* resistant to carbapenem and PB, and we did not measure prior use of antimicrobials other than meropenem and PB. It was not possible to analyze the adherence to infection control preventive measures (e.g., hand hygiene rate) during the study period. Finally, this is a single setting reality and may not be generalizable to other contexts.

In summary, COVID-19, coupled with an XDR-*K. pneumoniae* outbreak with high mortality rates, collapsed the local healthcare system not only in the COVID ICU but in different clinical settings in the hospital. In addition, the high labor demand may have favored the cross-transmission of this XDR *K. pneumoniae*, increasing antimicrobial consumption during the COVID-19 pandemic.

## Figures and Tables

**Table 1:** *K. pneumoniae* isolates susceptibility pattern and mutated genes related to PB resistance.

Period	Clinical Setting	Date	ID	ST	PB MIC (µg/mL)		Mutated Genes Related to PB Resistance
					Policimbac	Broth Microdilution	
September 2020	COVID ICU	14	L14	258	≥64	16	non identified
		14	L03	258	32	64	*mgrB*, *pmrA*
		14	L13	11	≥64	32	*mgrB*, *pmrA*, *pmrD*
		17	L11	258	32	32	*pmrA*
		18	L10	258	16	nd	*mgrB*, *pmrA*
		20	L30	258	≥64	≤0.5	*pmrA*, *pmrC*
		21	L09	258	≥64	32	*mgrB*, *pmrA*
		21	L05	258	4	8	*pmrA*
		25	L31	258	≥64	8	*pmrA*
		30	L15	258	≥64	4	*pmrA*
	Non-COVID ICU	14	L01	258	8	16	*pmrA*
		24	L06	258	≥64	32	*pmrA*
	Clinical Ward	5	L12	258	≥64	2	*pmrA*
	Surgical Ward	14	L02	258	64	256	*pmrA*
		17	L04	11	8	≤0.5	*pmrA*, *pmrD*, *ramA*
October 2020	COVID ICU	1	L34	437	≥64	32	*pmrA*, *pmrD*, *ramA*
		29	L18	258	≥64	64	*pmrA*
	Non-COVID ICU	5	L33	258	64	8	*pmrA*
		29	L16	258	32	4	*pmrA*
	Clinical Ward	8	L27	258	4	2	*pmrA*
		24	L24	258	4	≤0.5	*pmrA*
		3	L26	11	≥64	128	*mgrB*, *pmrA*, *pmrD*
	Surgical Ward	10	L28	11	16	256	*mgrB*, *pmrA*, *pmrD*
		29	L17	11	≥64	64	*mgrB*, *pmrA*, *pmrD*, *pmrK*
November 2020	COVID ICU	7	L19	16	32	16	*pmrA*, *pmrC*, *pmrD, pmrH*, *pmrI*, *pmrJ*, *pmrK, pmrL*, *ramA*
	Clinical Ward	5	L20	258	32	8	*mgrB*, *pmrA*

ICU: intensive care units; ID: sample identification; ST: sequence type; PB: polymyxin B; MIC: minimal inhibitory concentration.

**Table 2:** Demographic characteristics of 26 patients with positive culture for polymyxin B- and carbapenem-resistant *K. pneumoniae* in the period of September to November 2020.

Demographics Characteristics	Results
**Mean age (Min-Max)**	**57.8 (21–85)**
**Gender (*n* = 26)**	***n* (%)**
Male	15 (57.6)
Female	11 (42.3)
**Admission Unit *(n* = 26)**	***n* (%)**
COVID ICU	13 (50.2)
Non-COVID ICU	4 (15.3)
Clinical Ward	5 (19.2)
Surgical Ward	4 (15.3)
**Hospitalization days median (max-min)**	**49 (110–8)**
**Comorbidities (*n* = 26)**	***n* (%)**
Systemic Arterial Hypertension (SAH)	10 (40)
Diabetes Mellitus (DM)	4 (16)
Obesity	2 (8)
Other diseases	9 (36)
**Healthcare-associated infections (*n* = 18)**	***n* (%)**
Bloodstream infection	6 (33.3)
Ventilator-Associated Pneumonia	5 (27.8)
Urinary Tract Infection	3 (16.6)
Surgical Site Infection	2 (11.1)
Peritonitis	1 (5.6)
Tracheitis	1 (5.6)
**COVID-19 infection**	***n* (%)**
Positive	19 (73)
Negative	6 (23)
Inconclusive	1 (4)
**Passage by the intensive care unit *n* (%)**	**21 (80.7)**
**Pronation *n* (%)**	**7 (26.9)**
**Previous use of polymyxin *n* (%)**	**13 (50)**

## Data Availability

Not applicable.

## Supplementary Materials

The following supporting information can be downloaded at: https://www.mdpi.com/article/10.3390/antibiotics11060814/s1, Figure S1: Heatmap showing the presence of ARGs identified by ARG-ANNOT indicating the number of each resistance gene per genome. Data were clustered using hierarchical mapping with Euclidian distance. Blue to red scale indicates number of ARG for each strain in each category, as indicated in the legend. The nucleotide sequences in ARG-ANNOT from different antibiotics are abbreviated as follows: AGly, aminoglycosides resistance genes; Bla, beta-lactamases; Fcyn, fosfomycin; Flq, fluoroquinolones; Gly, glycopeptides; MLS, macrolide-lincosamide-streptogramin; Phe, phenicols; Rif, rifampin; Sul, sulfonamides; Tet, tetracyclines; and Tmt, trimethoprim. Bacteria holding plasmid pkP98M3N42 with coverage scores over 90% and similarity scores over 99% with the reference are indicated with a green solid circle; Figure S2: Number of *Klebsiella pneumoniae* isolates resistant to polymyxin and carbapenem in the Ribeirão Preto Clinics Hospital in 2019, 2020 and 2021 (Jan-Aug). Many patients had more than one isolate identified with the same drug-susceptibility profile. In 2021 there were 3.5 times more drug-resistant isolates than in 2019. During 2020 and 2021 the increment was quite related to the healthcare system collapse due to COVID-19 pandemic; Table S1: Antibiotic resistance profile of 26 PB- and carbapenem-resistant K. pneumoniae isolated from clinical patients during COVID-19 outbreak; Table S2: Acquired genes conferring resistance to beta-lactams, tetracyclines, aminoglycosides, fosfomycin, and fluoroquinolones found in the isolates genome.
