# Supplementary material for: Nosocomial Outbreak of Extensively Drug-Resistant (Polymyxin B and Carbapenem) Klebsiella pneumoniae in a Collapsed University Hospital Due to COVID-19 Pandemic
Source: Antibiotics (Basel). 2022 Jun 17;11(6):814. doi: 10.3390/antibiotics11060814 (PMC9219971; doi:10.3390/antibiotics11060814)
Supplement: Supplementary file 1 [file antibiotics-11-00814-s001.zip › antibiotics-1757867-supplementary.pdf]

Nosocomial Outbreak of Extensively Drug-Resistant (Polymyxin B and Carbapenem) *Klebsiella pneumoniae* in a Collapsed University Hospital Due to COVID-19 Pandemic

Gilberto G. Gaspar [1,*,†], Gustavo Tamasco [1,†], Nathália Abichabki [2,†], Ana Flavia T. Scaranello [3], Maria Auxiliadora-Martins [4], Renata Pocente [4], Leonardo N. Andrade [2], María-Eugenia Guazzaroni [3], Rafael Silva-Rocha [1], Valdes R. Bollela [1]

[1] Ribeirão Preto School of Medicine (FMRP), University of São Paulo (USP), Av. Bandeirantes 3900, Ribeirão Preto, SP 14049-900, Brazil gustavo.tamasco@usp.br (G.T.); silvarochar@usp.br (R.S.-R.); vbollela@fmrp.usp.br (V.R.B.)

[2] School of Pharmaceutical Sciences of Ribeirão Preto (FCFRP), University of São Paulo (USP), Av. Bandeirantes 3900, Ribeirão Preto, SP 14049-900, Brazil; nathalia.abichabki@alumni.usp.br (N.A.); leonardo@fcfrp.usp.br (L.N.A.)

[3] Faculty of Philosophy, Sciences and Letters of Ribeirão Preto (FFCLRP), University of São Paulo (USP), Av. Bandeirantes 3900, Ribeirão Preto, SP 14049-900, Brazil; anatonelli@usp.br (A.F.T.S.); meguazzaroni@ffclrp.usp.br (M.-E.G.)

[4] Clinics and University Hospital from Ribeirão Preto School of Medicine (FMRP), University of São Paulo (USP), Av. Bandeirantes 3900, Ribeirão Preto, SP 14049-900, Brazil; mamartins@hcrp.usp.br (M.A.-M.); rhcpocente@hcrp.usp.br (R.P.)

* Correspondence: ggaspar@hcrp.usp.br; Tel.: +55-(16)-981272799

† These authors contributed equally to this work.

Supplementary Methods

Parameter used for genome assemble and evaluation

Bowtie2 was used to align sequencing reads to long reference sequences with the following parameters: -D 20 -R 3 -N 1 -L 20 very-sensitive-local. The resulting reads were assembled using Spades with parameter -k 21, 33, 57, 77, 99, 127 -merge. After the annotation step performed by Prokka, all genes were aligned to NDARO using Diamond with the parameters blastx -k 5 -f 6 -Evalue 0.001.

Supplementary Tables

Table S1. Antibiotic resistance profile of 26 PB- and carbapenem-resistant *K. pneumoniae* isolated from clinical patients during COVID-19 outbreak

Strain ID	PB	TGC	AMP	SAM	PTZ	CRX	CRO	CAZ	CPM	ATM	ERT	IPM	MER	CZA	GEN	AMI	CL	CIP	SXT
L01	16,0	1,0	>32	>32	>128	>64	>64	16,0	>64	>32	>8	>16	>16	0.75	>16	>64	>64	>4	>320
L02	256,0	1,0	>32	>32	>128	>64	>64	>64	>64	>32	>8	>16	>16	1,0	>16	>64	>64	>4	>16:304
L03	64,0	1,0	>32	>32	>128	>64	>64	>64	>64	>32	>8	>16	>16	1,0	>16	>64	>64	>4	>320
L04	≤0,5	>8	>32	>32	>128	>64	>64	>64	>64	>32	>8	2,0	8,0	nd	≤1	8	>64	>4	>16:304
L05	8,0	2,0	>32	>32	>128	>64	>64	>64	>64	>32	>8	>16	>16	1,0	>16	>64	>64	>4	>16:304
L06	32,0	2,0	>32	>32	>128	>64	>64	16,0	>64	>32	>8	8,0	>16	1,0	>16	>64	>64	>4	>16:304
L09	32,0	2,0	>32	>32	>128	>64	>64	>64	>64	>32	>8	>16	>16	1,0	>16	>64	>64	>4	>16:304
L10	nd	nd	>32	>32	>128	>64	>64	>64	>64	nd	>8	>16	>16	nd	>16	>64	nd	>4	nd
L11	32,0	2,0	>32	>32	>128	>64	>64	>64	>64	>32	>8	>16	>16	0.016	>16	>64	>64	>4	>16:304
L12	2,0	0.5	>32	>32	>128	>64	>64	>64	>64	>32	>8	>16	>16	256,0	>16	>64	>64	>4	>16:304
L13	32,0	1,0	>32	>32	>128	>64	>64	>64	>64	>32	>8	1,0	4,0	nd	>16	32	64	>4	>16:304
L14	16,0	2,0	>32	>32	>128	>64	>64	32,0	>64	>32	>8	>16	>16	1,0	>16	>64	>64	>4	>16:304
L15	4,0	2,0	>32	>32	>128	>64	>64	>64	>64	>32	>8	>16	>16	1,0	>16	>64	>64	>4	>16:304
L16	4,0	4,0	>32	>32	>128	>64	>64	>64	>64	>32	>8	>16	>16	1,0	>16	>64	>64	>4	>16:304
L17	64,0	8,0	>32	>32	>128	>64	>64	>64	>64	>32	>8	>16	>16	S	>16	8	32	>4	>16:304
L18	64,0	2,0	>32	>32	>128	>64	>64	>64	>64	>32	>8	>16	>16	1.5	>16	>64	>64	>4	>16:304
L19	16,0	2,0	>32	>32	>128	>64	>64	>64	>64	>32	>8	>16	>16	S	≤1	4	16	>4	>16:304
L20	8,0	1,0	>32	>32	>128	>64	>64	>64	>64	>32	>8	>16	>16	S	>16	>64	>64	>4	>16:304
L24	<=0,5	2,0	>32	>32	>128	>64	>64	>64	>64	>32	>8	>16	>16	0.5	>16	>64	>64	>4	>16:304
L26	128	4,0	>32	>32	>128	>64	>64	>64	>64	>32	>8	>16	>16	0.25	≤1	16	32	>4	>16:304
L27	2	2,0	>32	>32	>128	>64	>64	>64	>64	>32	>8	>16	>16	1.5	>16	>64	>64	>4	>16:304

L28	256	1,0	>32	>32	>128	>64	>64	>64	>64	>32	>8	>16	>16	1.5	>16	16	64	>4	>16:304
L30	<=0,5	0.5	>32	>32	>128	>64	>64	>64	>64	>32	>8	>16	>16	0.5	>16	>64	>64	>4	>16:304
L31	8	2,0	>32	>32	>128	>64	>64	>64	>64	>32	>8	>16	>16	1,0	>16	>64	>64	>4	>16:304
L33	8	2,0	>32	>32	>128	>64	>64	>64	>64	>32	>8	>16	>16	0.75	>16	>64	>64	>4	>16:304
L34	32	4,0	>32	>32	>128	>64	>64	>64	>64	>32	>8	>16	>16	1,0	>16	16	16	>4	>16:304

Minimal inhibitory concentration is indicated in µg/mL. Red indicates resistance to the antibiotic, orange indicates susceptibility with increased exposure to antibiotic (previously intermediate category), and green indicates susceptible to antibiotic. Nd: not determined; S: susceptible, determined by disk-diffusion method; PB: Polymyxin B; TGC: Tigecycline; AMP: Ampicillin; SAM: Ampicillin+Sulbactam; PTZ: Piperacillin+Tazobactam; CRX: Cefuroxime; CRO: Ceftriaxone; CAZ: Ceftazidime; CPM: Cefepime; ERT: Ertapenem; IPM: Imipenem: MER: Meropenem; CZA: Ceftazidime/Avibactam; GEN: Gentamicin; AMI: Amikacin; NAL: Nalidixic acid; NOR: Norfloxacin; CIP: Ciprofloxacin; SXT: Trimethoprim Sulfamethoxazole.

Table S2. Acquired genes conferring resistance to beta-lactams, tetracyclines, aminoglycosides, fosfomycin, and fluoroquinolones found in the isolates genome.

Strain ID	Acquired resistance genes				
	**Beta-lactams (*bla*)**	**Tetracyclines**	**Aminoglycosides**	**Fosfomicyn**	**Fluoroquinolones**
L14	KPC-2; SHV-11; CTX-M-14	*tetG; tetR(G)*	*rmtB; aac3-IId; aac3-Ib; aadA2*	nd	nd
L03	KPC-2; SHV-11; CTX-M-14	*tetG; tetR(G)*	*rmtB; aac3-IId; aac3-Ib; aadA2*	nd	nd
L13	SHV-11; CTX-M-15	nd	*aac3-IIa; aac3-Ib; ant3''Ih-aac6-IId; aac3-Ib; aac3-Ib*	nd	*qnrB1*
L11	KPC-2; SHV-11; CTX-M-14	*tetG; tetR(G)*	*rmtB; aac3-IId; aac3-Ib; aadA2*	nd	nd
L10	KPC-2; SHV-11; CTX-M-14	*tetG; tetR(G)*	*rmtB; aac3-IId; aac3-Ib; aadA2*	nd	nd
L30	KPC-2; SHV-11; CTX-M-14	*tetG; tetR(G)*	*rmtB; aac3-IId; aac3-Ib; aadA2*	nd	nd
L09	KPC-2; SHV-11; CTX-M-14	*tetG; tetR(G)*	*rmtB; aac3-IId; aac3-Ib; aadA2*	nd	nd
L05	KPC-2; SHV-11; CTX-M-14	*tetG; tetR(G)*	*rmtB; aac3-IId; aac3-Ib; aadA2*	nd	nd
L31	KPC-2; SHV-11; CTX-M-14	*tetG; tetR(G)*	*rmtB; aac3-IId; aac3-Ib; aadA2*	nd	nd
L15	KPC-2; SHV-11; CTX-M-14	*tetG; tetR(G)*	*rmtB; aac3-IId; aac3-Ib; aadA2*	nd	nd
L01	KPC-2; SHV-11; CTX-M-14	*tetG; tetR(G)*	*rmtB; aac3-IId; aac3-Ib; aadA2*	nd	nd
L06	KPC-2; SHV-11; CTX-M-14	*tetG; tetR(G)*	*rmtB; aac3-IId; aac3-Ib; aac6-Ib; aadA2*	nd	nd
L12	KPC-2; SHV-11; CTX-M-14	*tetG; tetR(G)*	*rmtB; aac3-IId; aac3-Ib; aadA2*	nd	nd
L02	KPC-2; SHV-11; CTX-M-14	*tetG; tetR(G)*	*rmtB; aac3-IId; aac3-Ib; aadA2*	nd	nd
L04	SHV-11; CTX-M-66; CTX-M-15	nd	*ant3''Ih-aac6-IId; aac3-Ib*	nd	nd
L34	KPC-2; SHV-11; CTX-M-15	*tetG; tetR(G)*	*aac3-Ib; ant3''Ih-aac6-Iid; aadA2*	nd	*qnrB1*
L18	KPC-2; SHV-11; CTX-M-14	*tetG; tetR(G)*	*rmtB; aac3-IId; aac3-Ib; aadA2*	nd	nd
L33	KPC-2; SHV-11; CTX-M-14	*tetG; tetR(G)*	*rmtB; aac3-IId; aac3-Ib; aadA2*	nd	nd
L16	KPC-2; SHV-11; CTX-M-14	*tetR(G); tetG*	*rmtB; aac3-IId; aac3-Ib; aadA2*	nd	nd
L27	KPC-2; SHV-11; CTX-M-14; CTX-M-8	*tetG; tetR(G)*	*rmtB; aac3-IId; aac3-Ib; aac6-Ib; aadA2*	nd	*qnrB42*
L24	KPC-2; SHV-11; CTX-M-14	*tetG; tetR(G)*	*rmtB; aac3-IId; aac3-Ib; aadA2*	nd	nd
L26	KPC-2; SHV-11; CTX-M-15	nd	*ant3''Ih-aac6-IId; aadA2*	nd	*qnr-S1*
L28	KPC-2; SHV-11; CTX-M-15; CTX-M-14; CTX-M-8	*tetG; tetR(G); tetD*	*rmtB; aac3-IId; aac3-Ib; aac6-Ib; aadA1; aadA2; ant3''Ih-aac6-IId; aac6Ib-cr*	nd	*qnrB1; qnrB42*
L17	KPC-2; SHV-11; CTX-M-15	*tetR; tetA*	*aac3-Ib; ant3''Ih-aac6-IId; aac3-IIa; aadA2*	nd	nd

L19	KPC-2; SHV-145; CTX-M-15	*tetR; tetA*	*aac3-Ib; ant3''Ih-aac6-Iid; aadA2*	*fosA*	nd
L20	KPC-2; SHV-11; CTX-M-14	*tetG; tetR(G)*	*rmtB; aac3-IId; aac3-Ib; aadA2*	nd	nd

nd: not determined

Supplementary Figures

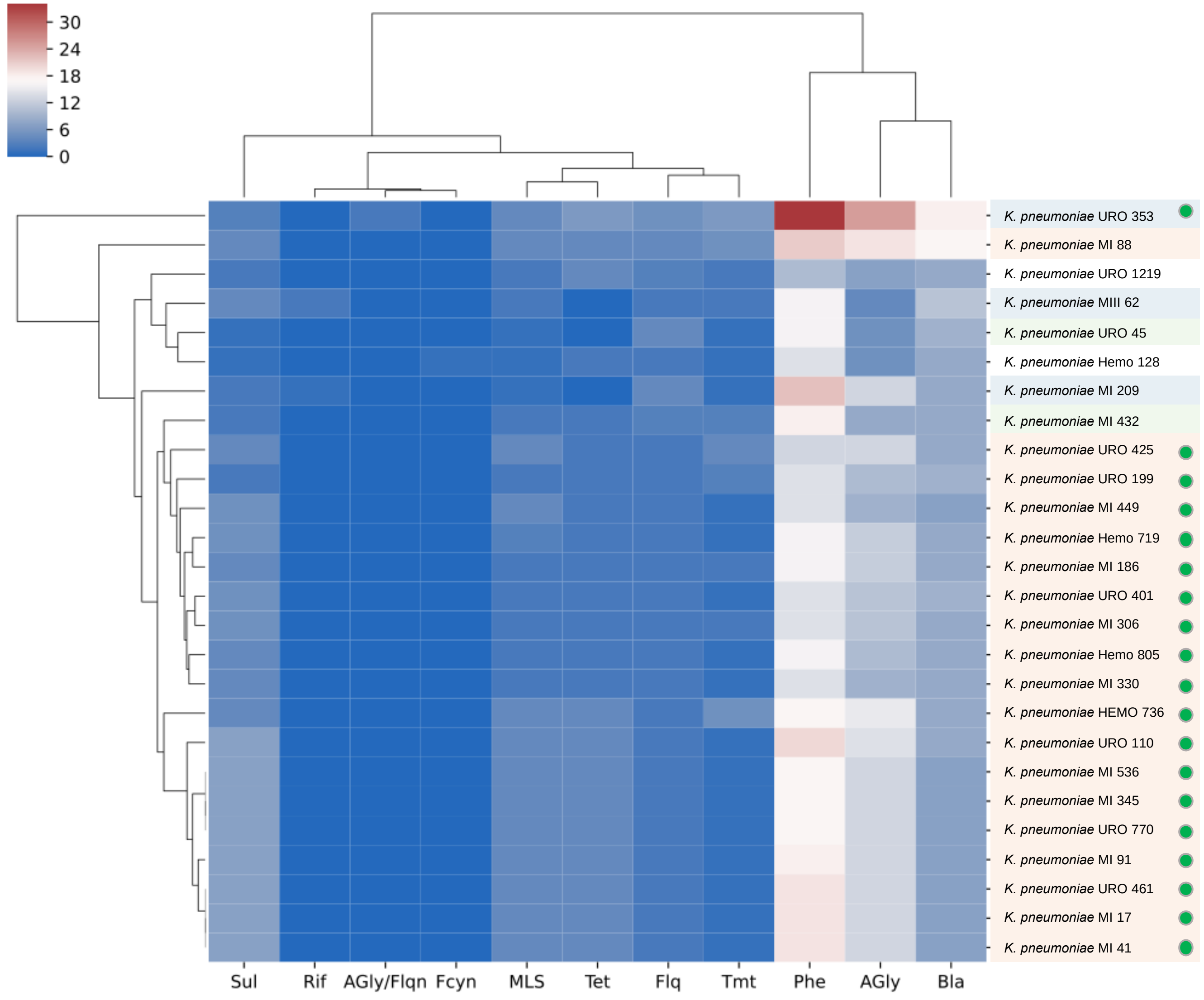

Figure S1. Heatmap showing the presence of ARGs identified by ARG-ANNOT indicating the number of each resistance gene per genome. Data were clustered using hierarchical mapping with Euclidian distance. Blue to red scale indicates number of ARG for each strain in each category, as indicated in the legend. The nucleotide sequences in ARG-ANNOT from different antibiotics are abbreviated as follows: AGly, aminoglycosides resistance genes; Bla, beta-lactamases; Fcyn, fosfomycin; Flq, fluoroquinolones; Gly, glycopeptides; MLS, macrolide-lincosamide-streptogramin; Phe, phenicols; Rif, rifampin; Sul, sulfonamides; Tet, tetracyclines; and Tmt, trimethoprim. Bacteria holding plasmid pkP98M3N42 with coverage scores over 90% and similarity scores over 99% with the reference are indicated with a green solid circle.

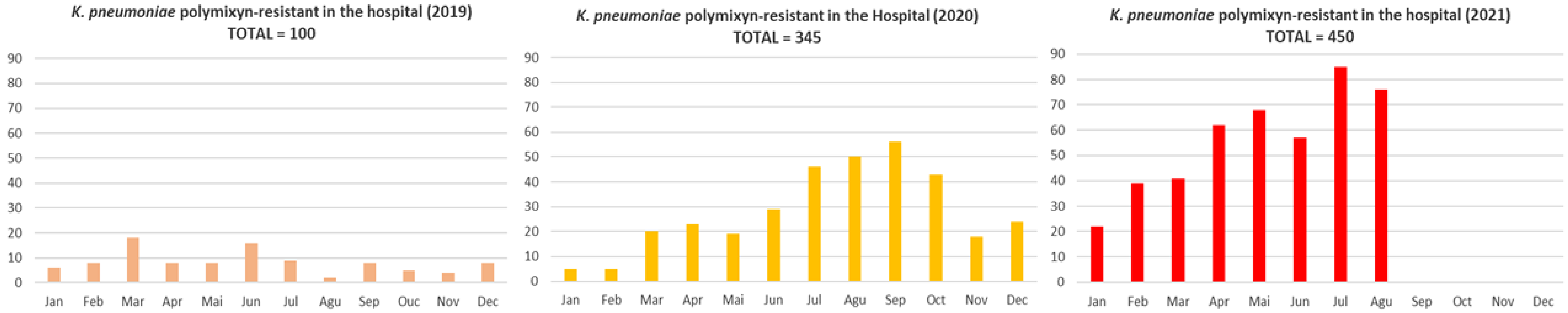

Figure S2. Number of *Klebsiella pneumoniae* isolates resistant to polymyxin and carbapenem in the Ribeirão Preto Clinics Hospital in 2019, 2020 and 2021 (Jan-Aug). Many patients had more than one isolate identified with the same drug-susceptibility profile. In 2021 there were 3.5 times more drug-resistant isolates than in 2019. During 2020 and 2021 the increment was quite related to the healthcare system collapse due to COVID-19 pandemic.
